# UAV Trajectory Tracking Using Proportional-Integral-Derivative-Type-2 Fuzzy Logic Controller with Genetic Algorithm Parameter Tuning

**DOI:** 10.3390/s24206678

**Published:** 2024-10-17

**Authors:** Oumaïma Moali, Dhafer Mezghani, Abdelkader Mami, Abdelatif Oussar, Abdelkrim Nemra

**Affiliations:** 1 UR-LAPER, Faculty of Sciences of Tunis, University of Tunis El Manar, Tunis 2092, Tunisia; dhafer.mezghani@fst.utm.tn (D.M.); abdelkader.mami@fst.utm.tn (A.M.); 2 School of Control and Automation, Ecole Militaire Polytechnique, EMP, Bordj El Bahri, Algiers 16111, Algeria; oussarabdelatif@gmail.com (A.O.); karim_nemra@yahoo.fr (A.N.)

**Keywords:** quadrotor, Type-1 FLC, Type-2 FLC, backstepping controller, robustness analyses, parameter uncertainties, genetic algorithm, wind gust

## Abstract

Unmanned Aerial Vehicle (UAV)-type Quadrotors are highly nonlinear systems that are difficult to control and stabilize outdoors, especially in a windy environment. Many algorithms have been proposed to solve the problem of trajectory tracking using UAVs. However, current control systems face significant hurdles, such as parameter uncertainties, modeling errors, and challenges in windy environments. Sensitivity to parameter variations may lead to performance degradation or instability. Modeling errors arise from simplifications, causing disparities between assumed and actual behavior. Classical controls may lack adaptability to dynamic changes, necessitating adaptive strategies. Limited robustness in handling uncertainties can result in suboptimal performance. Windy environments introduce disturbances, impacting system dynamics and precision. The complexity of wind modeling demands advanced estimation and compensation strategies. Tuning challenges may necessitate frequent adjustments, posing practical limitations. Researchers have explored advanced control paradigms, including robust, adaptive, and predictive control, aiming to enhance system performance amidst uncertainties in a scientifically rigorous manner. Our approach does not require knowledge of UAVs and noise models. Furthermore, the use of the Type-2 controller makes our approach robust in the face of uncertainties. The effectiveness of the proposed approach is clear from the obtained results. In this paper, robust and optimal controllers are proposed, validated, and compared on a quadrotor navigating an outdoor environment. First, a Type-2 Fuzzy Logic Controller (FLC) combined with a PID is compared to a Type-1 FLC and Backstepping controller. Second, a Genetic Algorithm (GA) is proposed to provide the optimal PID-Type-2 FLC tuning. The Backstepping, PID-Type-1 FLC, and PID-Type-2 FLC with GA optimization are validated and evaluated with real scenarios in a windy environment. Deep robustness analysis, including error modeling, parameter uncertainties, and actuator faults, is considered. The obtained results clearly show the robustness of the optimal PID-Type-2 FLC compared to the Backstepping and PID-Type-1 FLC controllers. These results are confirmed by the numerical index of each controller compared to the PID-type-2 FLC, with 12% for the Backstepping controller and 51% for the PID-Type-1 FLC.

## 1. Introduction

Using robots in search and rescue (SAR) operations has become a significant issue for a variety of applications. Search and rescue missions, as well as simulations, have revealed a number of areas where robot contributions could be improved. The quadrotor system is one of the most efficient and complicated robots, and it is widely employed in military and commercial applications. Autonomous navigation of a quadrotor (stability, trajectory tracking, obstacle avoidance, etc.) remains a difficult problem to solve.

**Figure 1:**
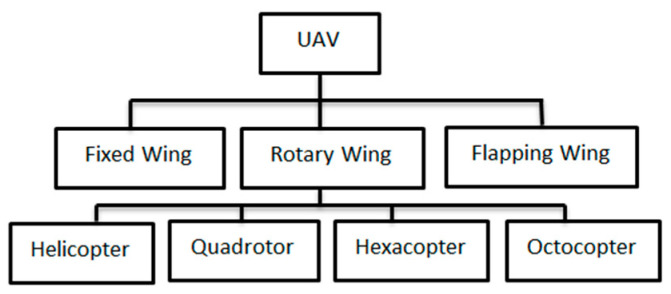
UAV classification.

In recent year, UAVs have known greater popularity for their applications due to inexpensive operating costs and high performance. They have been used in several fields, including surveillance and reconnaissance, battle assessment, and aerial photography. As depicted in Figure 1, a quadrotor is a multi-rotor micro aerial vehicle that is lifted and powered by four rotors. Quadrotor guidance, navigation, and control have become an active area of research among the versatile flying robotic platforms due to their exceptional rotational agility, mechanical simplicity, relatively small size, Vertical Take-Off and Landing (VTOL) ability, and affordability [1]. Furthermore, because it is unmanned, UAV operation eliminates all expenses and hazards connected with onboard human pilots in both civilian and military domains, such as landscape mapping, agricultural surveying, monitoring, and aerial photography [2].

The originality of this work consists of optimizing FLC Type-1 and Type-2 as well as backstepping controllers using a genetic algorithm, to improve their control robustness for quadrotor trajectory tracking in an uncertain environment. Three controllers are implemented and compared: the PID-Type-1 FLC, the PID-Type-2 FLC, and the backstepping controller with genetic algorithm optimization. First, a Type-2 FLC combined with a PID is compared to a PID-Type-1 FLC and Backstepping controller. Second, a GA is proposed to provide the optimal PID-Type-2 FLC tuning. The Backstepping controller, PID-Type-1 FLC, and PID-Type-2 FLC with GA optimization are validated and evaluated with real scenarios in a windy environment. Deep robustness analysis, including error modeling, parameter uncertainties, and actuator faults, is considered.

The related works are studied in the next section, followed by the system modeling with robustness analysis using the Wind Gust Model in Section 3. The different control designs are presented in Section 4, while Section 5 presents the results and discussion. The conclusions are given in Section 6.

## 2. Related Works

Several research works for quadrotor guidance and navigation have been proposed in the literature in the last few years. We recognize that crafting a high-quality flight design poses a significant challenge for quadrotor UAVs, given their nonlinear characteristics. In enhancing UAV flight performance, numerous researchers have introduced sophisticated methods to stabilize attitude and improve UAV positions and tracking. Several methods for automatic flight control systems have been considered for quadrotor localization, planification, and navigation. The Backstepping controller, which was used recently in [3], is widely used for UAV navigation [4]. An extension of adaptive Backstepping using neural networks is proposed in [5], where a Stable Adaptive Neural Control is proposed. Lee and Tomizuka designed a similar method using an FLC [6]. On the other hand, Pan et al. extended the use of an adaptive Backstepping approach for systems in strict-feedback form based on a multi-layer neural network [7]. Raziyeh et al. proposed a robust controller based on Backstepping and an Extended Kalman Bucy filter in [8]. Many researchers [9,10] have studied UAV navigation using FLCs. A novel PID FLC for quadrotor navigation using iterative learning control (ILC) was designed for a quadrotor unmanned aerial vehicle (UAV) in [11]. In the same case in [12], a hybrid PID-Fuzzy controller was studied for autonomous UAV stabilization. The Type-2 Fuzzy-PID approach is presented in [13] for the transmission line follower problem through UAVs. Nevertheless, the controllers previously proposed remain conventional in the face of the inherent complexity of the system and the constraints arising from uncertain parameters. This is where advanced research comes into play, aiming to enhance basic adaptive controllers through novel approaches, particularly by combining and optimizing these control parameters. Various new approaches have been proposed for system stabilization.

In the study referenced in [14], the authors employed a Fuzzy-Gain scheduling mechanism to fine-tune the PID controller for stabilizing both position and altitude. It is imperative for this control strategy to be efficacious, straightforward, and resilient against uncertainties and external disturbances. Reference [15] introduced the application of an Autonomous Quadcopter Trajectory Tracking and Stabilization system using a control system based on Sliding Mode Control and the Kalman Filter. Additionally, the authors of [16] proposed an algorithm for synthesizing an optimal controller to address the mixed H2/H∞ control problem for stabilizing aircraft during the glidepath landing mode under uncertainty. For example, in [17], the authors implemented a data-driven neuroendocrine-PID controller for underactuated systems. The notable advantage of this proposed approach lies in its capability to swiftly tune neuroendocrine-PID parameters by measuring the system’s input and output data without relying on a mathematical model.

In summary, several works have focused on the trajectory control of quadrotors. Thus, the comparison between numerous control methods makes it possible to demonstrate the robustness of the latter in an uncertain environment. This paper introduces various approaches, which can be classified into the following categories: model-based methods (using a Backstepping controller), model-free methods (employing Fuzzy Logic Controller Type-1 combined with PID and Fuzzy Logic Controller Type-2 Combined with PID), and parameter optimizations using genetic algorithms as a novelty.

## 3. System Modelling

### 3.1. Basic Concepts of Quadrotor UAVs

**Figure 2:**
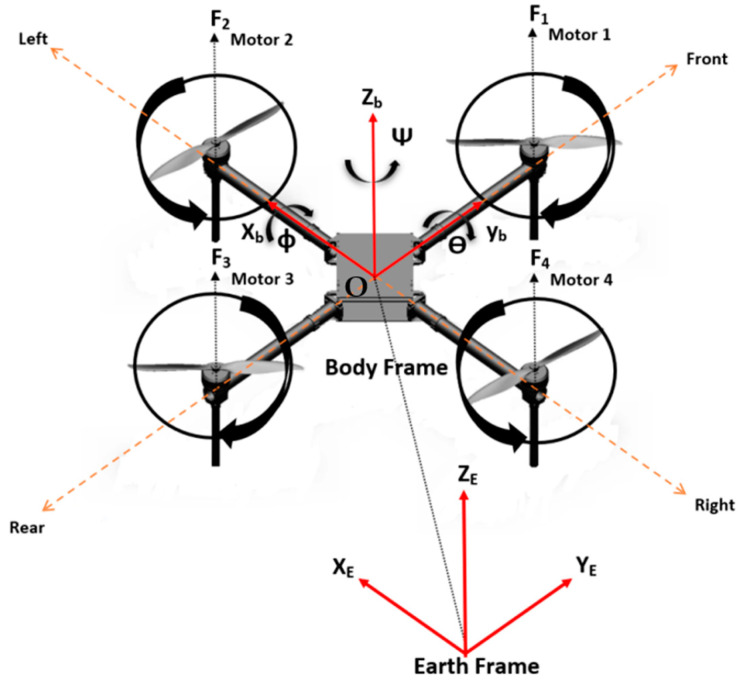
Quadrotor configuration [18].

The model of quadrotor illustrated in Figure 2 is taken from [18] and defined as a VTOL with four propellers. Its four propellers are placed at each extremity of a cross structure, where two pairs of propellers turn in opposite directions. The displacement or the rotation motions are performed by adjusting the angular velocity speeds of each rotor. By varying the rotor speeds, it is possible to make vertical lateral and longitudinal translations as well as a rotation about vertical axis. However, the quadrotor UAV has six Degrees of freedom (DOFs), three motions of rotations and translations in Cartesian space, and is controlled by only four inputs, which are thrust force, roll, pitch, and yaw moments.

### 3.2. Quadrotor Flight Kinematic and Dynamic Model

The quadrotor’s kinematics establish a connection between the vehicle’s position in the inertial frame and its velocity in the body frame. Meanwhile, the dynamics of the quadrotor describe the relationship between the applied body forces and the consequent accelerations.

#### 3.2.1. Kinematics

For Kinematic modeling, two frames are considered, as shown in Figure 2: the Earth Frame and the Body Frame. The relationship between the Earth Frame and the Body Frame is defined by the use of X, Y, and Z axes in the body-fixed frame, where O represents the center of mass. The X axis points toward rotor 1, the Y axis toward rotor 4, and the Z axis points upwards. The linear position and orientation of the Body Frame relative to the Earth Frame are expressed as three translations (X, Y, Z) and three Euler angles (roll, pitch, yaw-*φ*, *θ*, *ψ*). The linear velocities of the UAV in the body frame are denoted by (U, V, W), while (*p*, *q*, *r*) represent the angular rates with respect to the Body Frame. The rotation matrix between the Earth Frame and Body Frame is provided below (c: cos, s: sin).

(1) $$R=\left[\begin{array}{ccc} c\psi c\theta & -s\psi c\theta +c\psi s\theta s\varphi & s\psi s\varphi +c\psi s\theta c\varphi \\ s\psi c\theta & c\psi c\varphi +s\psi s\theta s\varphi & -c\psi s\varphi +s\psi s\theta c\varphi \\ -s\theta & c\theta s\varphi & c\theta c\varphi \end{array}\right]$$

By using the rotation matrix above, any point on the Body Frame can be expressed in the Earth Frame. An additional transformation matrix is required for the angular rates to transform the angular rates (p,q,r) in the body frame to the angular velocities $(\dot{\varphi },\dot{\theta },\dot{\psi })\;$in the inertial frame:

(2) $$R_{r}=\left[\begin{array}{ccc} 1 & 0 & -\sin\theta \\ 0 & \cos\varphi & \sin\varphi \cos\theta \\ 0 & -\sin\varphi & \cos\varphi \cos\theta \end{array}\right]$$

#### 3.2.2. Dynamics Model

**Figure 3:**
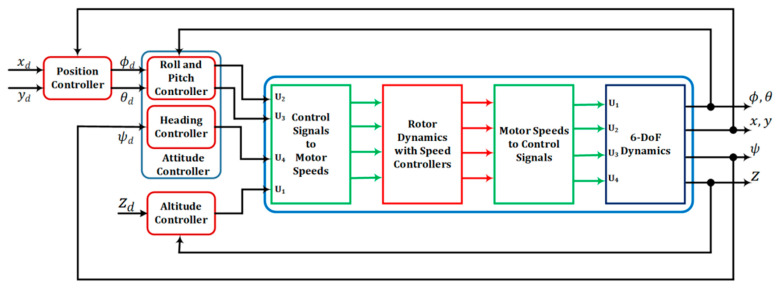
Control architecture of the quadrotor [21].

In this research, the quadrotor model is regarded as a rigid body with a concentrated point mass, featuring four rotors symmetrically positioned around its center of mass [19]. The control architecture of quadrotor is given by Figure 3. By employing Newton’s equations of translational and rotational motion, we can derive a set of six equations, as described below [1,20]:

(3) $$\begin{array}{l} \ddot{x}=(\sin\psi \sin\varphi +\cos\psi \sin\theta \cos\varphi )\;\frac{F_{z}}{m}+\frac{D_{x}}{m}\\ \ddot{y}=(-\cos\psi \sin\varphi +\sin\psi \sin\theta \cos\varphi )\frac{F_{z}}{m}+\frac{D_{y}}{m}\\ \ddot{z}=-g+(\cos\theta \cos\varphi )\;\frac{F_{z}}{m}+\frac{D_{z}}{m}\\ \ddot{\varphi }=\frac{\left(I_{yy}-I_{zz}\;\right)\dot{\theta }\dot{\psi }-J_{r}\dot{\theta }\omega_{r}+\tau_{x}}{I_{xx}}\;\\ \ddot{\theta }=\frac{\left(I_{zz}-I_{xx}\;\right)\dot{\varphi }\dot{\psi }+J_{r}\dot{\varphi }\omega_{r}+\tau_{y}}{I_{yy}}\\ \ddot{\psi }=\frac{(I_{xx}-I_{yy}\;)\dot{\varphi }\dot{\theta }+\tau_{z}}{I_{zz}} \end{array}$$

where ‘*m*’ signifies the mass of the quadrotor, and $I_{xx}$ $I_{yy}$, and $I_{zz}$ denote the inertial components along the x ,y, and z directions in frame B. The term $F_{z}$ represents the vertical thrust along the Z-axis. $\tau_{x}$, $\tau_{y}$, and $\tau_{z}$ represent the torques associated with the thrust difference of each rotor pair. Additionally, $D_{x}$, $D_{y}$, and $D_{z}$ indicate the drag forces corresponding to velocities in the X, Y, and Z directions. $J_{r}\;$shows the rotor inertia, and $\omega_{r}$ is the overall speed of the rotor, as defined below:

(4) $$\omega_{r}=-\omega_{1}+\omega_{2}-\omega_{3}+\omega_{4}$$

The quadrotor input controls are given by

(5) $$\begin{array}{l} u_{1}=b\left(\omega_{1}^{2}+\omega_{2}^{2}+\omega_{3}^{2}+\omega_{4}^{2}\right)\;\\ u_{2}=b\left(\omega_{4}^{2}-\omega_{2}^{2}\right)\\ u_{3}=b\left(\omega_{3}^{2}-\omega_{1}^{2}\right)\\ u_{4}=b(\omega_{2}^{2}+\omega_{4}^{2}-\omega_{1}^{2}-\omega_{3}^{2}) \end{array}$$

The relations between $F_{z}\;$, $\tau_{x},\;\tau_{y},\;\tau_{z}\;$and each rotor’s angular speeds can be obtained by analyzing the free body diagram of the model in Figure 2. The terms$\;\omega_{1}$, $\omega_{2}$, $\omega_{3},$ and $\omega_{4}$ represent the angular speeds of the four rotors, b represents thrust factor, d represents drag factor, and l represents the length of each quadrotor’s arm. The following matrix shows the relationships between $F_{z}\;$,$\tau_{x},\;\tau_{y},\;\tau_{z}$ and the angular velocities of the four propellers:

(6) $$\left[\begin{array}{c} F_{z}\\ \tau_{x}\\ \tau_{y}\\ \tau_{z} \end{array}\right]=\left[\begin{array}{cccc} b & b & b & b\\ 0 & -lb & 0 & lb\\ -lb & 0 & lb & 0\\ d & -d & d & -d \end{array}\right]\left[\begin{array}{c} w_{1}^{2}\\ w_{2}^{2}\\ w_{3}^{2}\\ w_{4}^{2} \end{array}\right]$$

The dynamic model has the potential to be simplified and expressed as a set of nonlinear dynamic equations, which can be characterized as follows:

(7) $$\ddot{X}=\left(X\right)+g\left(X\right)u$$

where u and X ϵ${\;R}^{4}$ are, respectively, the input and state vector, given as follows:

(8) $$u={\left[u_{1}u_{2}u_{3}u_{4}\right]}^{T}$$

(9) $$X={\left[X_{1}X_{2}X_{3}X_{4}\right]}^{T}={\left[z\varphi \theta \psi \right]}^{T}$$

The matrices representing the nonlinear dynamic function, denoted as *f*(*X*), and the nonlinear control function, denoted as *g*(*X*), can be written as follows:

(10) $$f\left(X\right)=\left(\begin{array}{c} -g\\ \dot{\theta }\dot{\psi }a_{1}-\dot{\theta }a_{2}\Omega_{d}\\ \dot{\theta }\dot{\psi }a_{3}+\dot{\varphi }a_{4}\Omega_{d}\\ \dot{\theta }\dot{\varphi }a_{5} \end{array}\right)\;g\left(X\right)=\left(\begin{array}{cccc} u_{z}\frac{1}{m} & 0 & 0 & 0\\ 0 & b_{1} & 0 & 0\\ 0 & 0 & b_{2} & 0\\ 0 & 0 & 0 & b_{3} \end{array}\right)$$

with the following abbreviations:

$$\begin{array}{l} a_{1}=(I_{yy}-I_{zz}/I_{xx}),\;a_{2}=J_{r}/I_{xx},\;a_{3}=(I_{ZZ}-I_{xx})/I_{yy},\;a_{4}=J_{r}/I_{yy},\;\;\\ a_{5}=(I_{xx}-I_{yy}/I_{zz}),\\ b_{1}=l/I_{xx},\;b_{2}=l/I_{yy},\;b_{3}=l/I_{zz},\;u_{z}=(\cos\varphi \cos\theta ). \end{array}$$

Given the well-known values of all system parameters, the nominal representation of nonlinear systems can be articulated as follows:

(11) $$\ddot{X}=f_{0}\left(X\right)+g_{0}\left(X\right)u$$

where $f_{0}$ and $g_{0}$ are the nominal value of f and g, respectively.

In our case, we are interested in the uncertainties in thrust and lift coefficients, as well as inertial moments, which pose challenges in accurately characterizing the behavior of quadrotors. These uncertainties can arise due to various factors. For thrust coefficients, changes in environmental conditions, such as temperature and air density, can affect thrust production.

In addition, environmental factors like wind gusts and turbulence can introduce variations in lift coefficients during flight, and surface contamination or damage to the airframe can impact lift generation.

The inertial moments depend on the distribution of mass within the quadrotor. Irregularities or asymmetry in mass distribution can lead to variations in the moments of inertia. In windy conditions, changes in the distribution of lift and drag forces on the vehicle may affect its rotational behavior. inertial moments manifest in different axes, corresponding to yaw, pitch, and roll motions. Wind-induced moments can lead to changes in the quadrotor’s orientation and rotation around these axes, affecting its overall stability.

### 3.3. Robustness Analysis Using Wind Gust Model

Wind gusts are intricate physical phenomena commonly represented using either deterministic models [22] or stochastic models [23]. The latter models assume that turbulence behaves as a stationary Gaussian random process, which is a prevalent assumption. The stationary nature implies that turbulence extends infinitely in duration, while the Gaussian process pertains to the probability of encountering a specific gust velocity at a given time. A stochastic approach employed in modeling wind gusts is the Power Spectral Density (PSD). In the PSD atmospheric turbulence model, it is presumed that the turbulence’s intensity is significantly influenced by various factors and can change due to weather conditions, flight altitude, and temperature gradients [24]. Wind gust signals are generated by passing white noise through a shaping filter. In the literature, two primary shaping filters can be identified: the Dryden filter and the Von Karman filter. Given its simpler form, we opt to use the Dryden filter in this study. The filters employed to generate the Dryden spectral model are provided in [25,26], as follows:

(12) $$H_{u}\left(s\right)=\frac{\Delta u}{N_{w}}=\sigma_{u}\sqrt{\frac{2L_{u}}{\pi v}}\frac{1}{1+\frac{L_{u}}{v}S}\;H_{v}\left(s\right)=\frac{\Delta v}{N_{w}}=\sigma_{v}\sqrt{\frac{L_{v}}{\pi v}}\frac{1+\frac{\sqrt{3Lv}}{v}S}{{\left(1+\frac{L_{v}}{v}S\right)}^{2}}\;H_{w}\left(s\right)=\frac{\Delta w}{N_{w}}=\sigma_{w}\sqrt{\frac{L_{w}}{\pi v}}\frac{1+\frac{\sqrt{3L_{w}}}{v}S}{{\left(1+\frac{L_{w}}{v}S\right)}^{2}}$$

where $N_{w}$ represents white noise, v signifies the relative velocity of the UAV quadrotor concerning the airflow, and [Δu, Δv, Δw]^T^ represents alterations in body linear velocities due to wind gusts. The turbulence intensities (σu, σv, σw) and the turbulence scale lengths (Lu, Lv, Lw) provide descriptions of the wind gust characteristics. In regions with low altitude (where the altitude is less than 1000 feet), the turbulence scale lengths and intensities are defined as follows:

(13) $$L_{w}=h,\;L_{u}=L_{v}=\frac{h}{{\left(0.177+0.000823h\right)}^{1.2}}$$

(14) $$\sigma_{w}=0.1w_{20},\frac{\sigma_{u}}{w}=\frac{\sigma_{v}}{w}=\frac{1}{{\left(0.177+0.000823h\right)}^{0.4}}$$

In this paper, we consider a standard wind speed of 2.4 m/s, which corresponds to Beaufort Scale 3, and an altitude of 2 m. Here, h represents the height above the ground, and W*_20_* denotes the wind speed as per the Beaufort Scale.

## 4. Control Design

**Figure 4:**
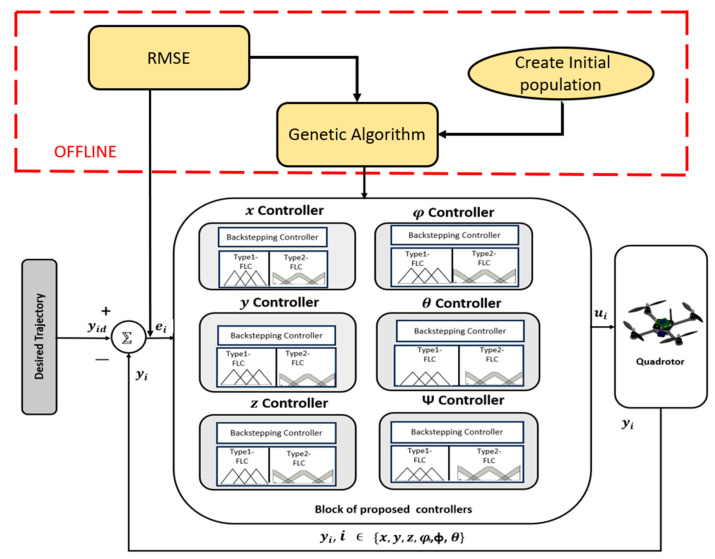
Global model proposed for controller system for quadrotor.

In this paper, two categories of controllers are investigated: model-based controllers and FLCs. For the former, a Backstepping controller is implemented and validated. For the latter, two FLCs are considered: Type 1 and Type 2. The control approaches with and without the model are implemented and validated on a trajectory tracking problem using a quadrotor. Figure 4 shows the global model proposed controllers’ system for a quadrotor.

### 4.1. Backstepping Controller

The control objective is to develop a suitable control law for the system [8], allowing the state vector *X* of the quadrotor system to adhere to a predetermined reference trajectory vector *Xd*. The following section provides an overview of the Backstepping control approach designed for the quadrotor system:

Step 1: First, the tracking error is defined.

(15) $$e_{1}=X_{d\;}-X$$

where $X_{d\;}$ is a desired trajectory specified by a reference model. Then, the derivative of the tracking error can be represented as

(16) $$\dot{e_{1}}=\dot{X_{d}}-\dot{X}$$

The first Lyapunov function is chosen as

(17) $$V_{1}\left(e_{1}\right)=\frac{1}{2}e_{1}^{T}e_{1}$$

The derivative of $V_{1}$ is

(18) $$\dot{V_{1}}\left(e_{1}\right)=e_{1}^{T}\dot{e_{1}}=e_{1}^{T}\left(\dot{X_{d}}-\dot{X}\right)$$

where $\dot{X}$ can be viewed as a virtual control. The desired value of virtual control α, known as a stabilizing function, can be defined as follows:

(19) $$\alpha =\dot{X_{d}}+k_{1}e_{1}$$

where $k_{1}$ is a positive constant. By substituting the virtual control by its desired value from Equation (17), we obtain

(20) $$\dot{V_{1}}\left(e_{1}\right)=-k_{1}e_{1}^{T}e_{1}\leq 0$$

Step 2: The deviation of the virtual control from its desired value can be defined as

(21) $$e_{2}=\dot{X}-\alpha =\dot{X}-\dot{X_{d}}-k_{1}e_{1}$$

The derivative of $e_{2}$ is expresses as

(22) $$\dot{e_{2}}=\ddot{X}-\dot{\alpha }=f_{0}\left(X\right)+g_{0}\left(X\right)u+L-\ddot{X_{d}}-k_{1}\dot{e_{1}}$$

The second Lyapunov function is

(23) $$V_{2}\left(e_{1},e_{2}\right)=\frac{1}{2}e_{1}^{T}e_{1}+\frac{1}{2}e_{2}^{T}e_{2}$$

The derivative equations of (23) are defined as

(24) $$\dot{{\;V}_{2}}\left(e_{1},e_{2}\right)=e_{1}^{T}\dot{e_{1}}+e_{2}^{T}\dot{e_{2}}=e_{1}^{T}\dot{{(X}_{d}}-\dot{X})+e_{2}^{T}(\ddot{X}-\dot{\alpha })=e_{1}^{T}\left(-e_{2}-k_{1}e_{1}\right)+e_{2}^{T}(f_{0}\left(X\right)+g_{0}\left(X\right)u+L-\ddot{X_{d}}-k_{1}\dot{e_{1}})=\;-k_{1}e_{1}^{T}e_{1}+e_{2}^{T}(-e_{1}+f_{0}\left(X\right)+g_{0}\left(X\right)u+L-\ddot{X_{d}}-k_{1}\dot{e_{1}}$$

Step 3: Since the system dynamics and the external disturbance are well known, and $g_{0}\left(X\right)\neq 0,\;$an ideal Backstepping can be obtained as

(25) $$u=g_{0}{\left(X\right)}^{-1}(e_{1}+k_{1}\dot{e_{1}}+\ddot{X_{d}}-f_{0}\left(X\right)-L-k_{2}e_{2}$$

where $k_{2}$ is a positive constant, and the term $k_{2}e_{2}$ is added to stabilize the tracking error. Substituting (25) into (24), the following equation can be obtained:

(26) $$\dot{V_{2}}\left(e_{1},e_{2}\right)=-k_{1}e_{1}^{T}e_{1}-k_{2}e_{2}^{T}e_{2}$$

where $\dot{V_{2}}\left(e_{1},e_{2}\right)\leq 0,\;\dot{V_{2}}\left(e_{1},e_{2}\right)$ is a negative semi-definite. So, the Backstepping controller in (25) will stabilize the system.

### 4.2. Type-1 Fuzzy Logic Controller

**Figure 5:**
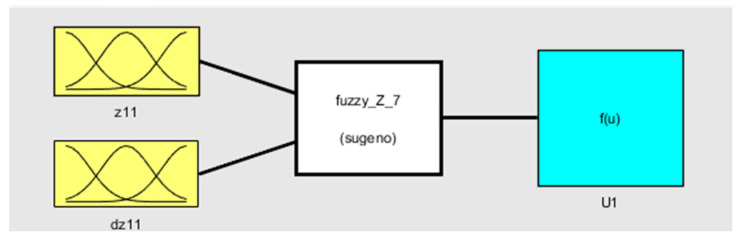
Type-1 fuzzy logic controller.

Fuzzy logic offers straightforward computational capabilities and is widely regarded as one of the most adaptable controllers. In this paper, first, a Takagi Sugeno Type-1 FLC controller is proposed for quadrotor trajectory tracking, as shown in Figure 5.

This paper introduces and compares four different variations for controlling roll, pitch, yaw, and height through simulation and careful observation.

**Figure 6:**
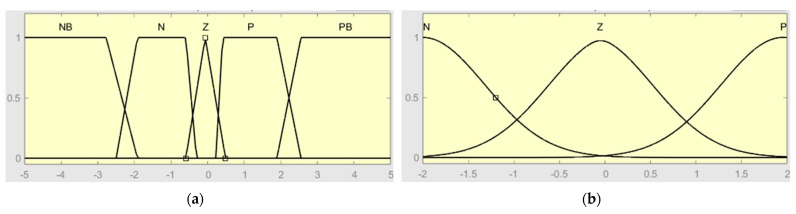
Membership function of Type-1-FLC: (**a**) first input (e); (**b**) second input (de).

The membership functions in Figure 6 are tuned based on the range sensors and the authors’ experience. Increasing the number of membership functions can provide good accuracy; however, the computational time will increase. Then, the shape and number of membership functions should be selected to maintain a suitable trade-off between precision, robustness, and computational time. Table 1, shows the quadrotor rule base.

For quadrotor control, the triangular, trapezoid, and Gaussian membership functions are used. The input range is [−2, 2], whereas the output variable lies in the range of [−15, 15]. The membership for each controller is as shown in Figure 6a,b. Table 2 presents the parameters of output.

### 4.3. Type-2 Fuzzy Logic Controller 

**Figure 7:**
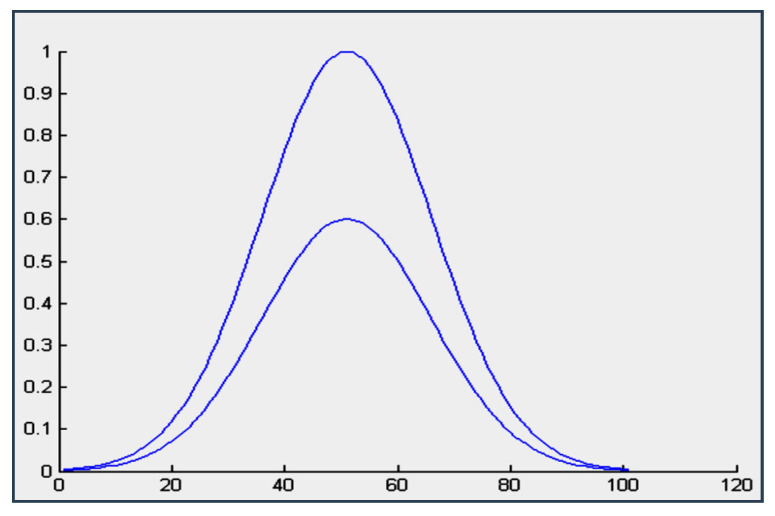
Model of membership function for Type-2 FLC.

A Type-2 fuzzy logic system or controller shares many fundamental concepts with a Type-1 FLC. In fact, a Type-2 fuzzy logic system closely resembles a Type-1 system in terms of membership functions, fuzzy rules, fuzzification, inference, and defuzzification [27]. There are only two key distinctions: firstly, in the Type-2 FLC, the membership functions are three-dimensional, as depicted in Figure 7. This third dimension represents the value of the membership function at each point within its two-dimensional domain, known as the footprint of uncertainty (FOU) [28]. Secondly, the Type-2 FLC necessitates an additional step involving type reduction.

**Figure 8:**
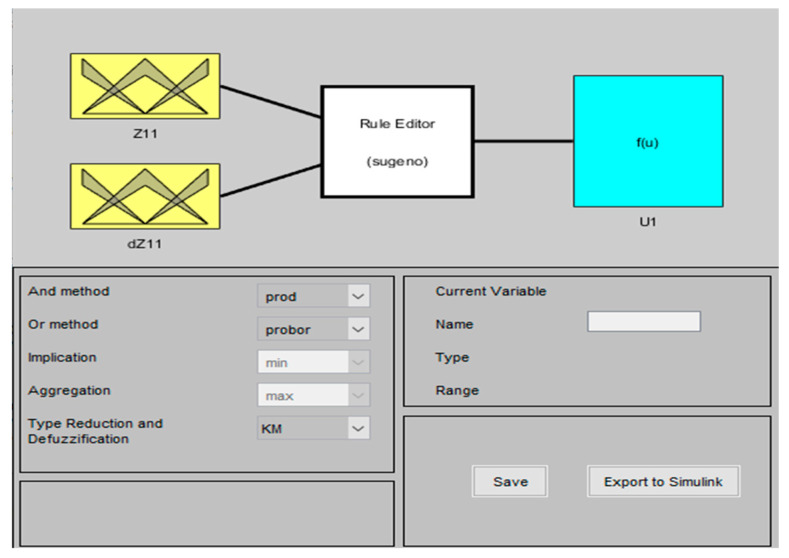
Type-2 fuzzy logic controller.

To create a Type-2 FLC, we expanded upon the initially suggested Type-1 controller by leveraging the IT2-FLS v1.1 Matlab/Simulink Toolbox (Figure 8). The inputs, rule base, and outputs closely mirror those of the Type-1 FLC discussed in Section 4.2.

**Figure 9:**
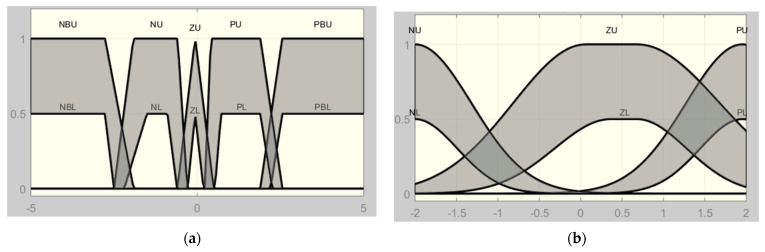
Membership function of Type-2-FLC: (**a**) first input (e); (**b**) second input (de).

The membership functions of the Type-2 fuzzy logic controller inputs are represented below (Figure 9a,b):

Similar to the approach taken for the Type-1 FLC, we propose a set of 15 IF-THEN fuzzy rules for the Type-2 FLC. The output processing block utilizes the Takagi–Sugeno “SOM/PROD” inference method and adopts the “NT” type for the reduction and defuzzification process.

### 4.4. PID-Fuzzy Logic Controller

The main drawback of the FLC approach is the tuning of membership parameters of input and output when a significant number of rules is considered. Using human expertise is efficient for a few number of rules. However, when the number of rules increases, it becomes very difficult to tune the FLC; thus, for the solution, we propose to use a PID-FLC optimized by a genetic algorithm.

### 4.5. PID-FLC with GA Tuning

**Figure 10:**
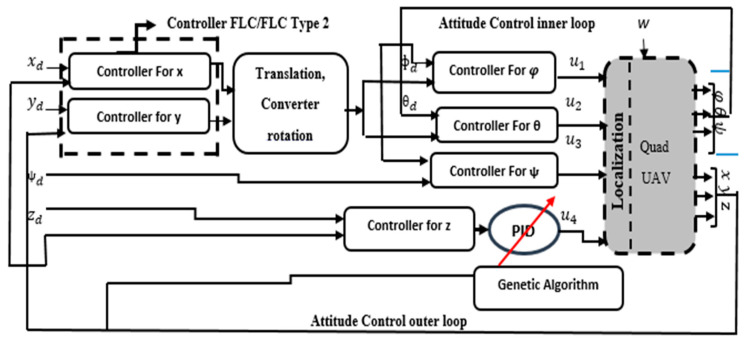
Quadrotor control scheme with GA optimization.

In the figure below (Figure 10), an overall summary diagram explains the principal of the optimal PID-Type-1 FLC controller and PID-Type-2 FLC controller based on GA parameter optimization. Red arrow indicates that the PID parameters are optimized by GA.

### 4.6. Optimal Type-1 and Type-2 FLC with PID Controller

The primary benefit of the FLC is its compatibility with a conventional PID controller. As the name implies, this control technique combines aspects of two control methods: fuzzy logic and PID [2].

The PID controller finds extensive application across various domains. Its merits include the elimination of steady-state errors, overshooting, reduction in settling time, and enhancement of system stability. The mathematical equation for the PID controller is presented below:

(27) $$u=K_{p}e+K_{i}\int_{0}^{t}e\;dt+K_{d}\frac{d}{dt}\;e$$

To achieve the desired output in the control of the quadrotor, a combination of three PD controllers and one PID controller is employed. Below, we provide a description of each controller:

(28) $$u_{1}=K_{p}\left(\varphi_{d}-\varphi \right)+K_{d}(\dot{\varphi_{d}}-\dot{\varphi )}\;\;{\;\;\;\;\;\;\;\;\;\;\;\;\;\;\;\;\;\;\;u}_{2}=K_{p}\left(\theta_{d}-\theta \right)+K_{d}(\dot{\theta_{d}}-\dot{\theta )}\;{\;\;\;\;\;\;\;\;\;\;\;\;\;\;\;\;\;\;\;u}_{3}=K_{p}\left(\psi_{d}-\psi \right)+K_{d}(\dot{\psi_{d}}-\dot{\psi )}\;u_{4}=K_{p}\left(z_{d}-z\right)+K_{i}\int_{0}^{t}z+K_{d}(\dot{z_{d}}-\dot{z)}$$

where $u_{1},u_{2},u_{3},{\mathrm{and}\;u}_{4}$ are the control inputs and $K_{p}$, $K_{d},$ and $K_{i}$ are, respectively, the proportional gain, the derivative gain, and the integral gain.

### 4.7. Genetic Algorithm

#### 4.7.1. Definition and Schemes

GAs are search processes that operate according to the principles of natural selection and genetics. A basic GA encompasses three key operations: Selection, Genetic Operations, and Replacement, as illustrated in the figure below, representing a typical GA cycle [25].

**Figure 11:**
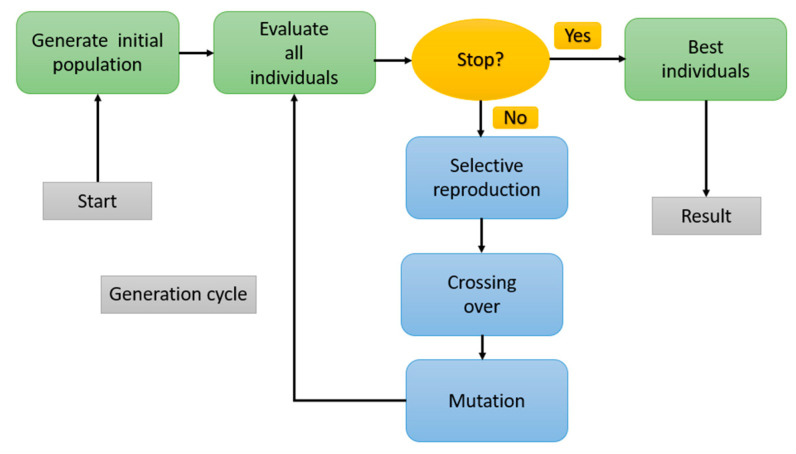
Generic algorithm cycle.

Genetic algorithms involve the evolutionary cycle of a series of genes (Figure 11), referred to as a chromosome; this symbolizes a potential solution to the problem at hand. Each gene within the chromosome corresponds to a component of the solution pattern. The prevalent method for representing a solution as a chromosome involves employing a string of binary digits, where each bit in the string acts as a gene. The transformation of the solution into the binary bit string is termed coding. The choice of a particular coding scheme is application-dependent. Subsequently, the solution bit strings undergo decoding to facilitate their evaluation through a fitness measure [29].

The motivation for employing a genetic algorithm in the control system of a quadrotor, in particular for the optimization of PID and FLC parameters, came from the fact that the latter exhibit complex, non-linear dynamics, making it difficult to determine optimal control parameters analytically [30]. In addition, GAs are highly effective in handling complex non-linear optimization problems, without requiring a detailed understanding of the system’s mathematical model; they are particularly robust in addressing uncertainties and adapt well to variations, providing a means to optimize control parameters for different operating conditions. On the other hand, PID and FLC controllers often involve setting several parameters to balance different control objectives, such as stability, responsiveness, and robustness. Thus, GAs support multi-objective optimization, enabling the exploration of a solution space that optimally balances these conflicting objectives. In addition, GAs are suitable for offline optimization, where the algorithm can iteratively explore the parameter space without the need for real-time computation [31].

There are many other optimization algorithms that are successfully applied to tune FLC parameters, such as the hybrid spiral bacteria search algorithm [32], the hybrid gray whale optimization approach [33], and many others. These approaches are good in terms of computation time, which is not really important in our case.

#### 4.7.2. Advantages and Disadvantages of Using GAs

The following table (Table 3) summarizes the advantages and disadvantages of using genetic algorithms in the control system optimization of a quadrotor as compared with stated algorithms such as the hybrid spiral-bacterial foraging algorithm and hybrid Grey Wolf–Whale optimization approach.

### 4.8. Optimization Strategy of Using GA

**Figure 12:**
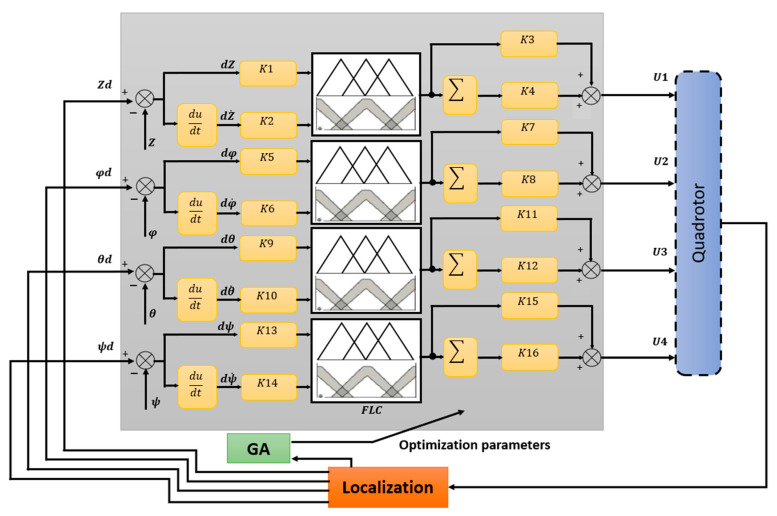
Architecture of optimization strategy for Type-1 FLC and Type-2 FLC using GA.

Efficiency in terms of the noisy or stochastic objective function is, in this paper, achieved using the proposed PID-Type-1 FLC as well as the PID-Type-2 FLC, automatically tuned using the genetic algorithm. The PID-Type-1 FLC and PID-Type-2 FLC with GA optimization are shown in Figure 12.

As can be seen from previous PID FLC controllers, including the Z-Controller, φ-Controller, θ-Controller, and ψ-Controller, sixteen (16) parameters should be tuned. For this purpose, a Genetic Algorithm (GA) was used to optimize these parameters in order to minimize the Root Mean Square Error (RMSE) of the trajectory tracking using the quadrotor. In this case, the fitness function can be given by

(29) $$f\left(K\right)=\sqrt{{\left(X_{d}-X\right)}^{2}+{\left(Y_{d}-Y\right)}^{2}+{\left(Z_{d}-Z\right)}^{2}}$$

where K=[K1,K2,K3,…,K16].

The optimization problem is solved with a genetic algorithm:

(30) ArgminK=1:12 ⁡Xd−X2+Yd−Y2+Zd−Z2

**Figure 13:**
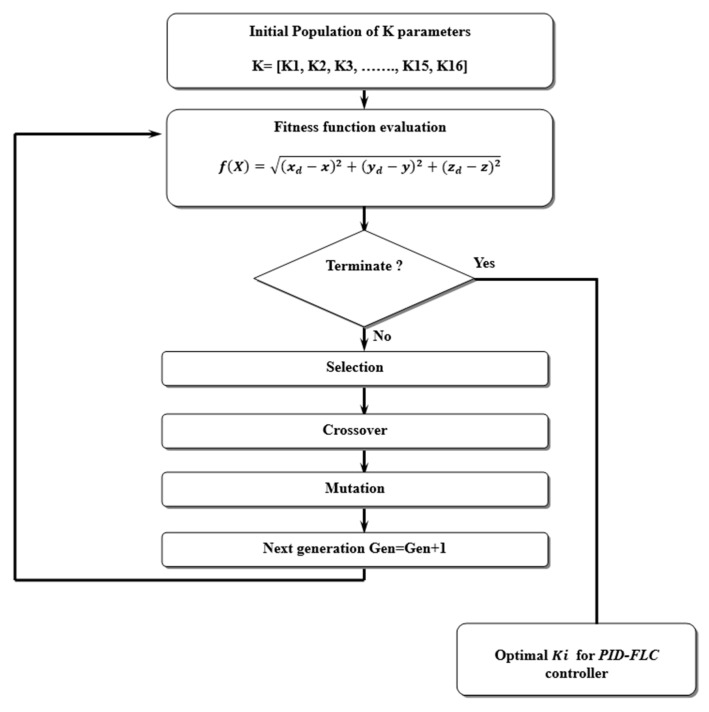
GA optimization step diagram.

GA optimization steps are given in the diagram below (Figure 13):

## 5. Results and Discussion

To evaluate the performances of the proposed controllers, many simulation scenarios are considered. The quadrotor parameters used for simulation are taken from [1] and are listed in Table 4.

**Figure 14:**
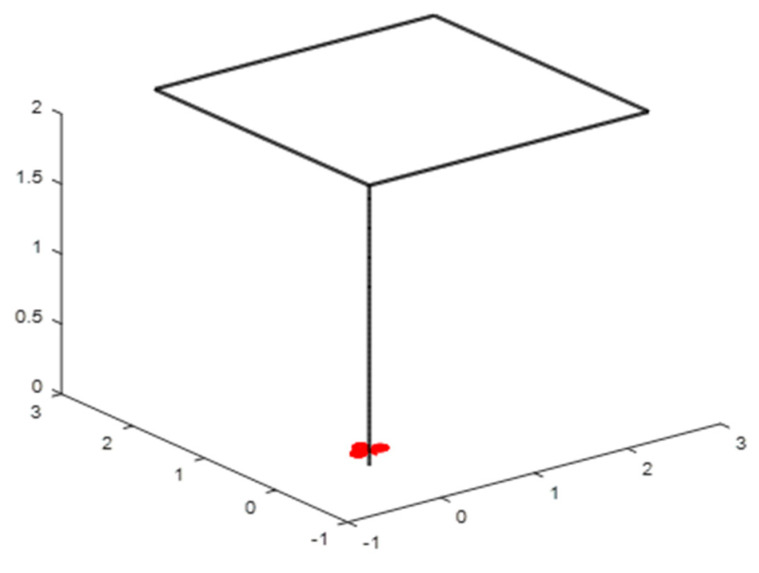
Trajectory of simulation.

The proposed algorithms are validated using a trajectory tracking scenario (Figure 14). The quadrotor (shown in red) should accurately follow the predefined trajectory. Three controllers are implemented, validated, and compared using realistic scenarios.

### 5.1. Path Tracking for Quadrotor Using Backstepping Controller

**Figure 15:**
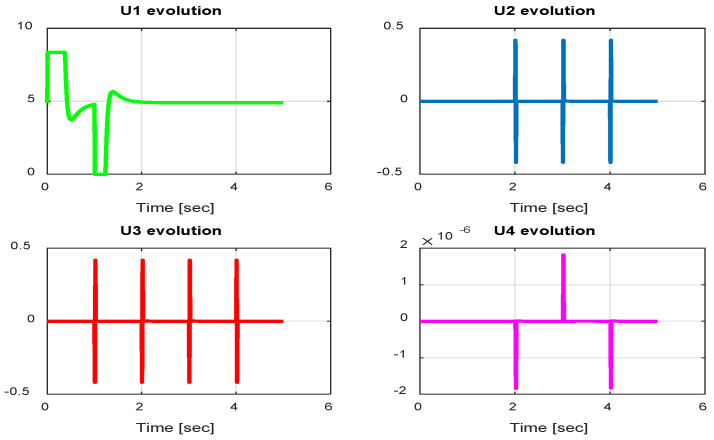
Quadrotor commands of Backstepping control.

**Figure 16:**
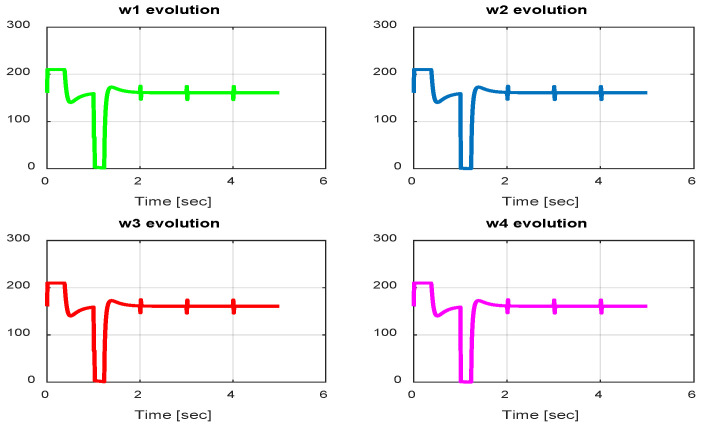
Motor velocities of Backstepping control.

The first step covers quadrotor trajectory tracking; the vehicle is initialized and then takes off from the initial position. The Backstepping controller is generally capable of piloting the quadrotor to the trajectory reference and stabilizing the altitude of the vehicle in a few seconds after takeoff, as shown in the figures below (Figure 15 and Figure 16).

**Figure 17:**
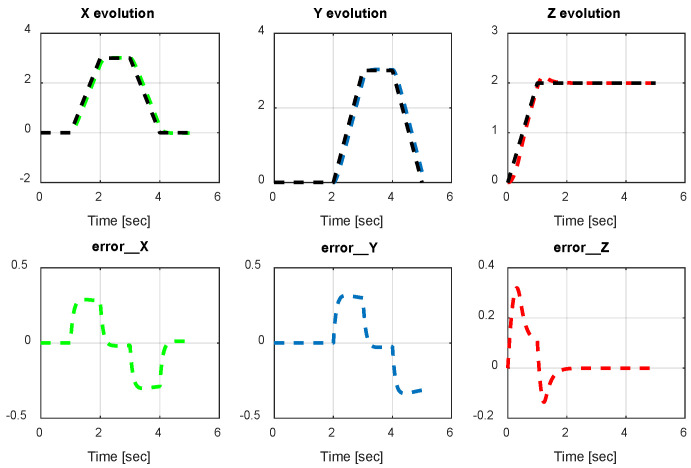
*x*, *y*, z errors evolution of Backstepping control.

**Figure 18:**
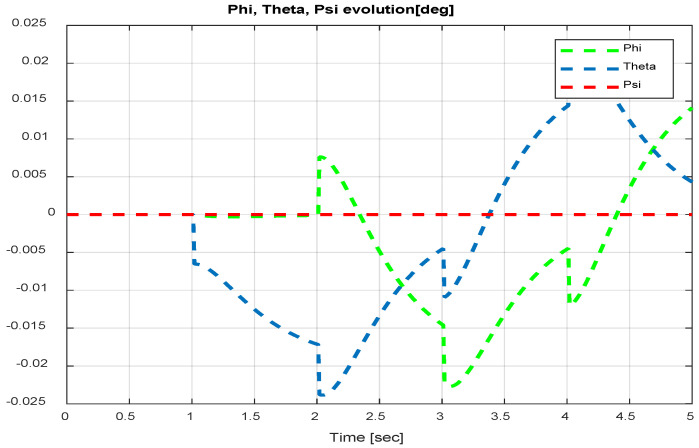
Quadrotor angles of Backstepping control.

The obtained results of the quadrotor trajectory tracking using the Backstepping controller are illustrated in Figure 15, Figure 16, Figure 17 and Figure 18. According to the obtained results, the UAV accurately follows the desired trajectory (see Section 5.4). This result is confirmed by Figure 17 and Figure 18, where very small errors following x, y, z and θ, φ, ψ are obtained. Figure 15 and Figure 16 illustrate the evolution of the velocity and input command of the quadrotor, respectively.

### 5.2. Path Tracking for Quadrotor Using Type-1 Fuzzy Logic Controller

The results of trajectory tracking using the Type-1 FLC are presented in this section. The first test illustrates the accurate tracking of the trajectory. The UAV is tested with different initial positions before being piloted autonomously by simulation. In addition, the quadrotor is tested using a trajectory that has a turn and a climb, with various initial positions, to observe the ability to follow a given trajectory. The figures below show the obtained results.

**Figure 19:**
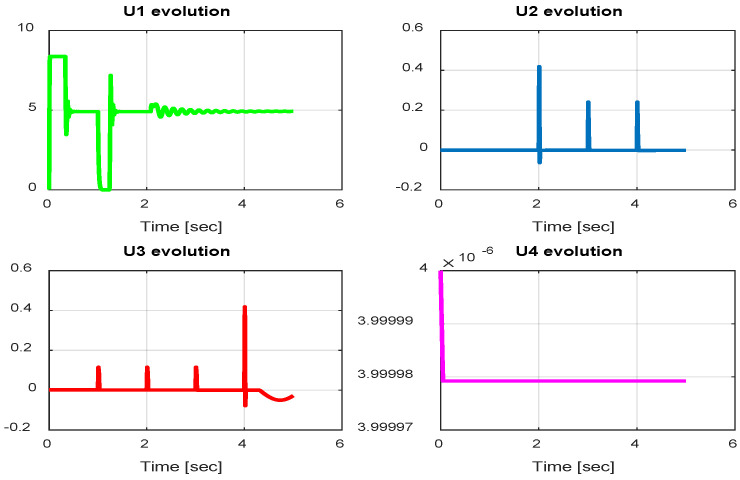
Quadrotor commands of Type-1 FLC.

**Figure 20:**
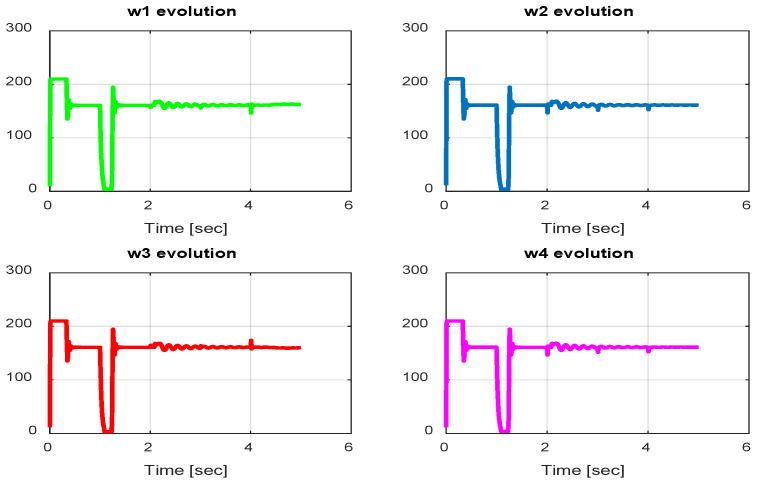
Motor velocities of Type-1 FLC.

**Figure 21:**
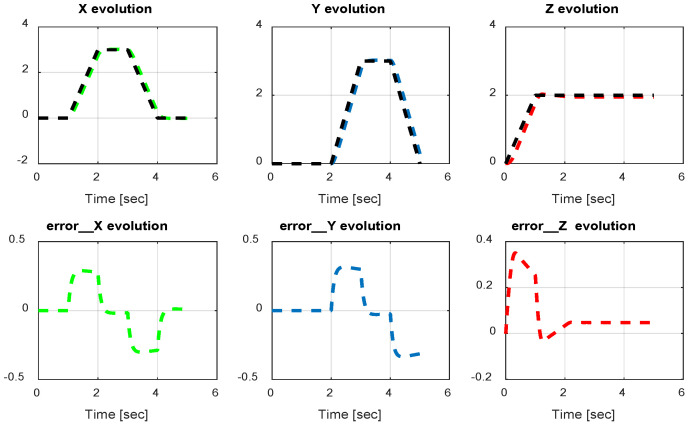
*x*, *y*, z error evolution of Type-1 FLC.

**Figure 22:**
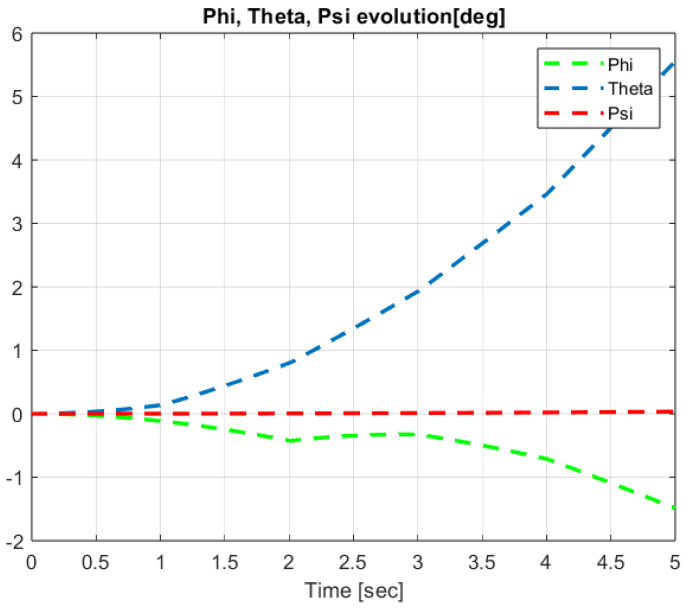
Quadrotor angles of Type-1 FLC.

The input response and velocity evolution provided by the Type-1 FLC are illustrated in Figure 19 and Figure 20, respectively. According to the results illustrated above for quadrotor navigation using the Type-1 FLC, the quadrotor accurately follows the desired trajectory (see Section 5.4). These results are confirmed by the small value of error given in Figure 21 and Figure 22.

### 5.3. Path Tracking for Quadrotor Using Type-2 Fuzzy Logic Controller

**Figure 23:**
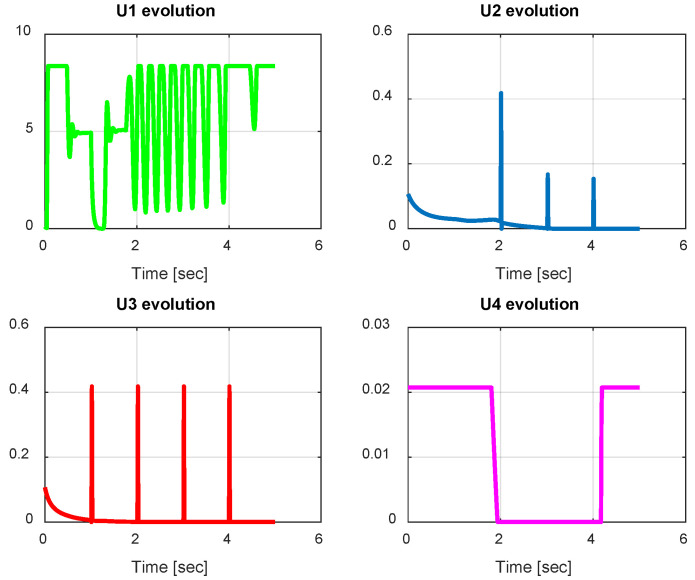
Quadrotor commands of Type-2 FLC.

**Figure 24:**
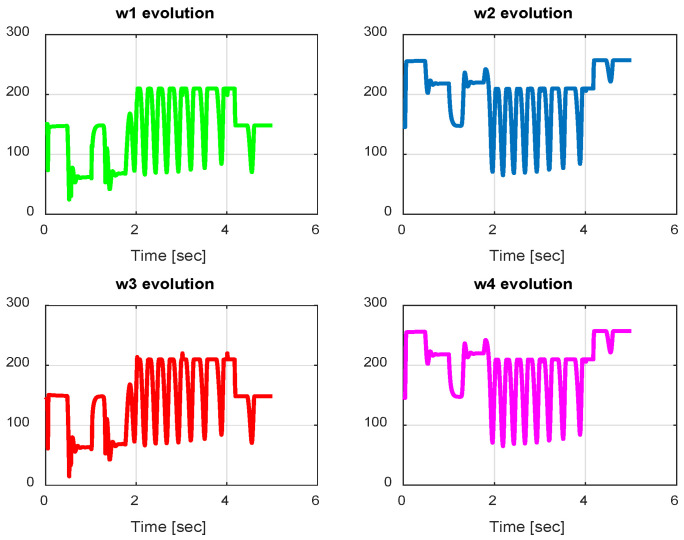
Motor velocities of Type-2 FLC.

**Figure 25:**
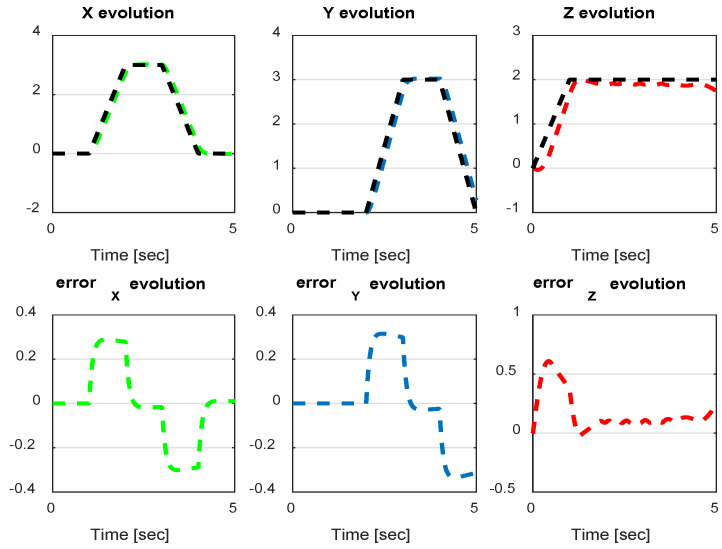
*x*, *y*, z error evolution of Type-2 FLC.

**Figure 26:**
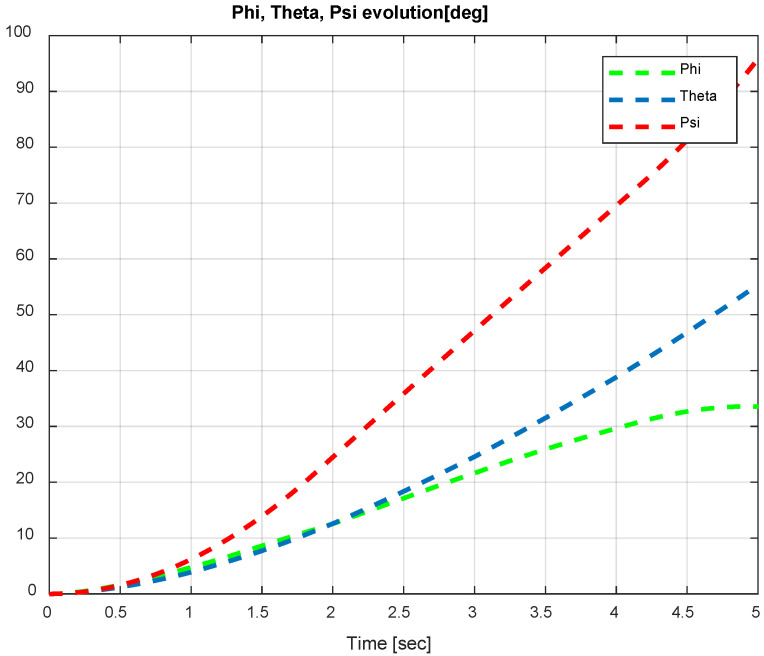
Quadrotor angles of Type-2 FLC.

**Figure 27:**
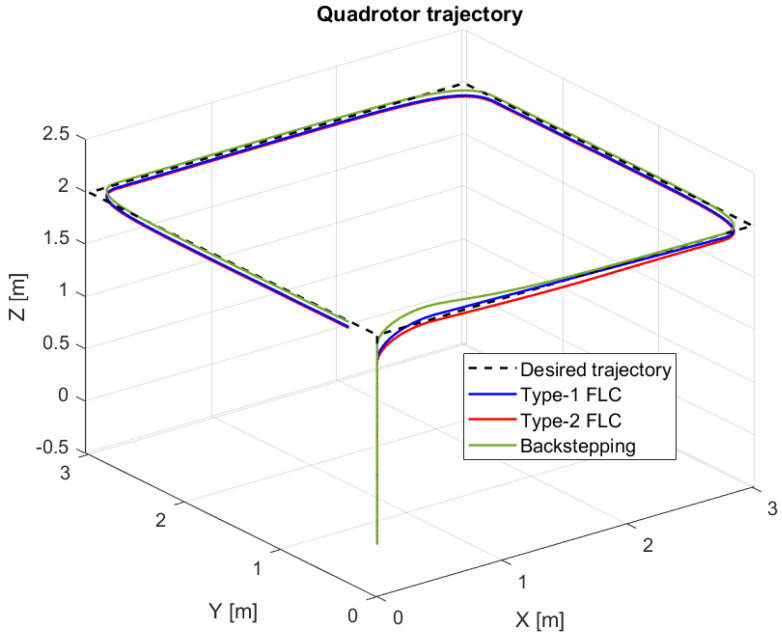
Quadrotor trajectory for proposed controllers.

In this section, we present simulation results to evaluate the Type-2 FLC in a trajectory tracking scenario. The proposed controller is validated according to the desired path navigation problem; the Type-2 FLC shows good performance even with the complexity of the quadrotor parameters, as can be seen in Figure 23, Figure 24, Figure 25, Figure 26 and Figure 27.

### 5.4. Comparison between Proposed Controllers (Backstepping, Type-1 FLC, and Type-2 FLC)

In this section, we compare the three controllers for trajectory tracking (Figure 27) to confirm the results given in the figures above for each model. The three controllers give quite similar results for trajectory tracking, with good accuracy.

### 5.5. Path Tracking for Quadrotor Using PID-Type-1 FLC and PID-Type-2 FLC Controllers with GA Tuning

The performance of the FLC controller is well known; it does not require any system modeling and is able to treat uncertainties. Furthermore, the FLC controller is robust and easy to implement. However, it can be difficult to accurately tune the fuzzy rules (in our case, 15 fuzzy rules). As a solution for this problem, and to ensure the quadrotor is able to track the predefined trajectory with high precision, PID-Type-1 FLC and PID-Type-2 FLC controllers are implemented, validated, and compared in this section. Moreover, to obtain optimal performance, the PID parameters are optimized using GA (Figure 12); the results of optimization are given in Table 5, with nine parameters tuned with GA.

#### 5.5.1. PID-Type-1 FLC with GA Tuning Results

**Figure 28:**
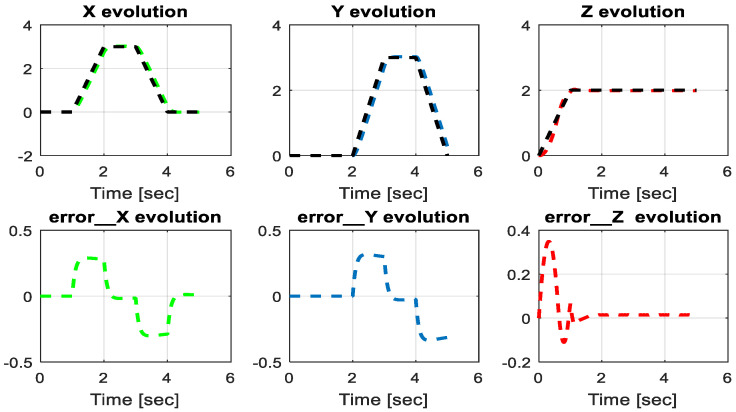
The errors of X, Y, and Z position using the PID-Type-1 FLC controller with GA.

**Figure 29:**
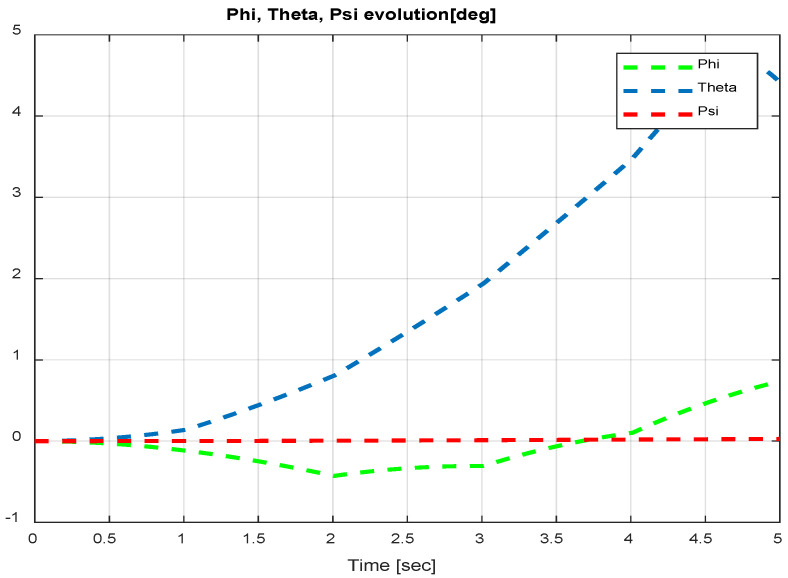
Quadrotor angles of PID-Type-1 FLC controller with GA.

**Figure 30:**
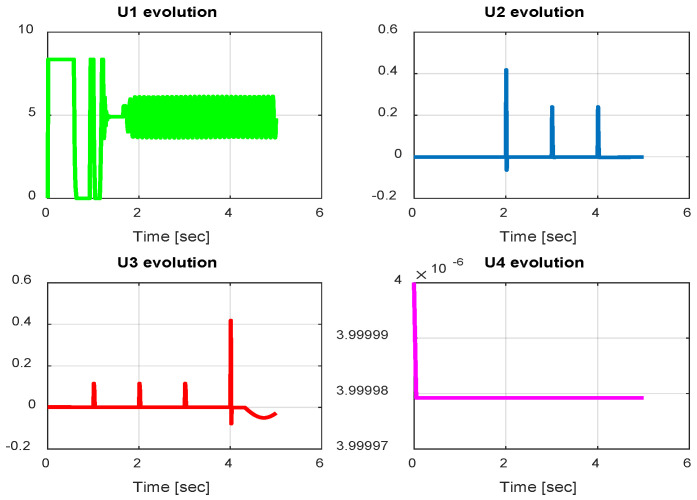
Quadrotor commands of PID-Type-1 FLC controller with GA.

**Figure 31:**
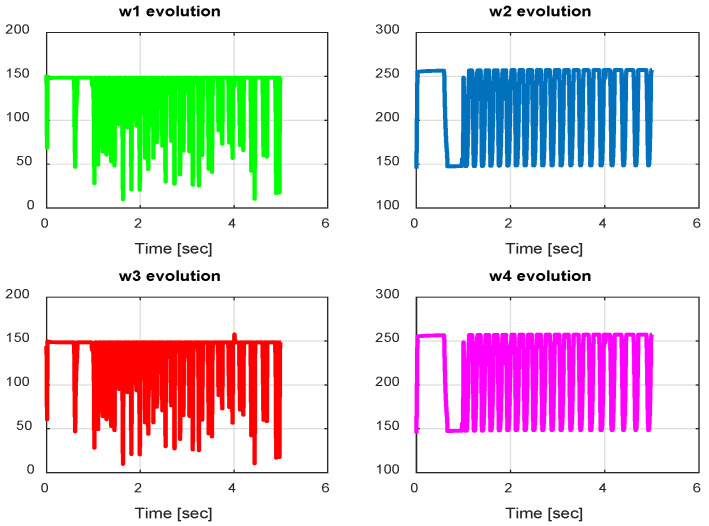
Motor velocities of PID-Type-1 FLC controller with GA.

From the simulation results, the *PID-Type-1 FLC* controller using GA optimization provides good performances for the trajectory tracking, as confirmed by Figure 28 and Figure 29. The position and orientation errors are illustrated, respectively, with small errors obtained. Figure 30 and Figure 31 illustrate the input controller (quadrotor command) and the velocity evolution. We notice some chattering in the input controller when using GA optimization.

#### 5.5.2. PID-Type-2 FLC with GA Tuning Results

**Figure 32:**
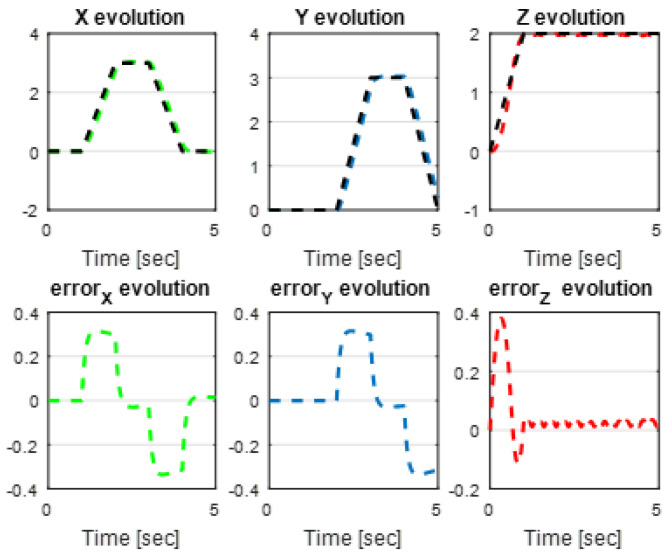
The errors of X, Y, and Z for the PID-Type-2 FLC controller with GA.

**Figure 33:**
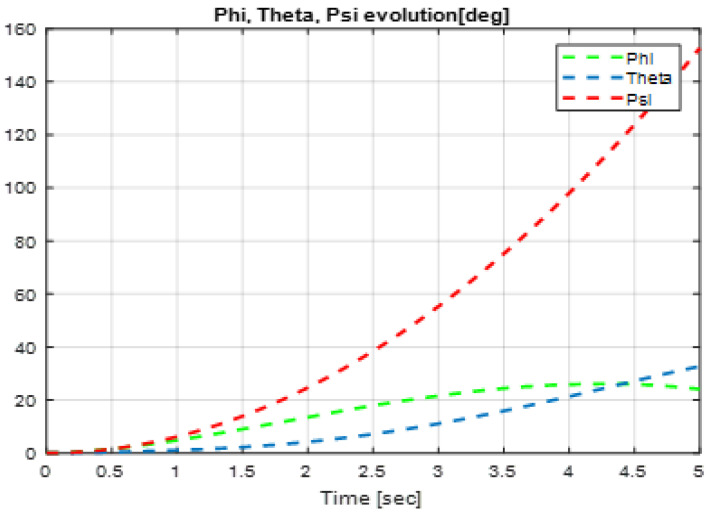
Quadrotor angles of PID-Type-2 FLC controller with GA.

**Figure 34:**
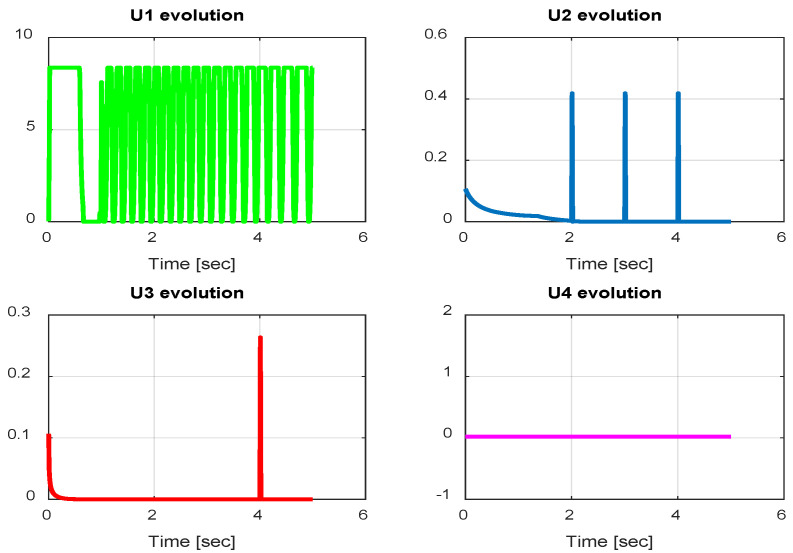
Quadrotor commands of PID-Type-2 FLC controller with GA.

**Figure 35:**
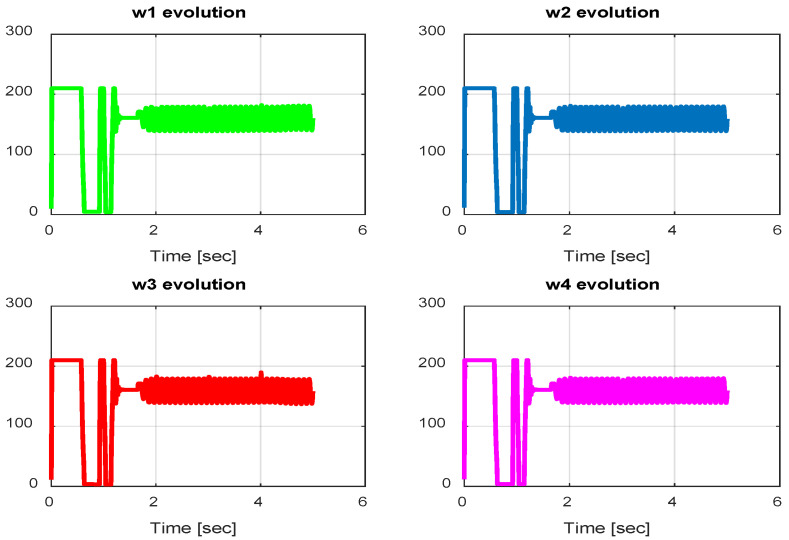
Motor velocities of PID-Type-2 FLC controller with GA.

**Figure 36:**
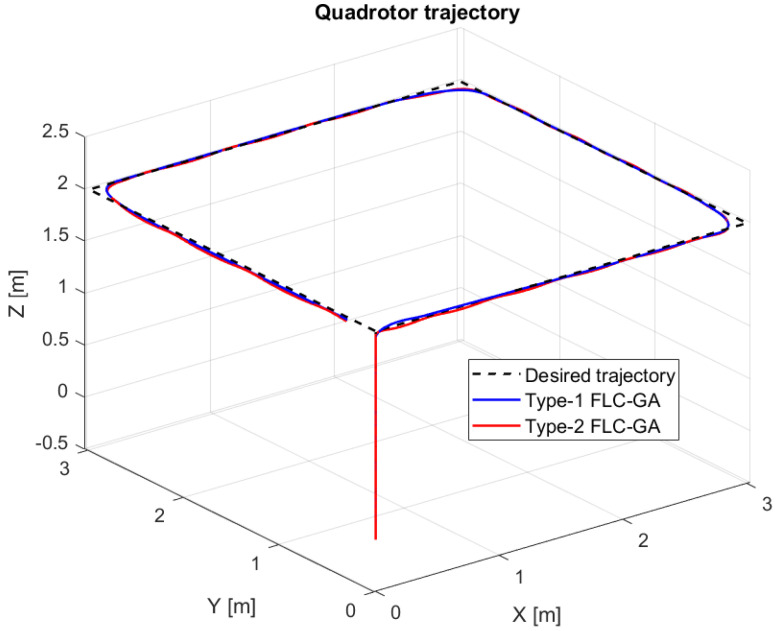
Quadrotor trajectory for PID-Type-1 FLC and PID-Type-2 FLC controllers with GA optimization.

In this section, we present simulation results to evaluate the proposed controller (PID-Type-2 FLC) with GA parameter tuning. Figure 32 and Figure 33 illustrate the pose errors (position and orientation, respectively). UAV commands and motor velocities are given, respectively, in Figure 34 and Figure 35; as can be seen, the chattering effect is reduced significantly with the Type-2 FLC controller. As shown in Figure 36, the Type-2 FLC maintains good performance.

Figure 36 illustrates the comparison between Type-1 FLC and Type-2 FLC controllers using GA optimization. The Type-2 FLC with GA optimization performs better than the Type-1 FLC controller, due to its robustness in optimizing these parameters.

### 5.6. Robustness Analysis

In this section, we consider a significant disturbance using a wind gust model. Simulations have been conducted to assess the robustness and efficacy of the proposed controller in tracking a specified trajectory. Two distinct simulation scenarios involving varying wind gusts are taken into account. The wind gust model outlined in Section 3 is employed to generate wind velocity along the three axes—lateral, longitudinal, and vertical. Subsequently, these velocities are treated as external disturbances affecting the translational velocity of the UAV [25].

In the first scenario, the wind gust model parameters are *Lu* = *Lv* = 23.568, *Lw* = 3, *σu* = *σv* = 0.48, and *σw* = 0.14, and a typical wind speed of 2.4 m/s is considered.

In the second scenario, the parameters of the wind gust model are *Lu* = *Lv* = 23.568, *Lw* = 3, *σu* = *σv* = 0.68, and *σw* = 0.34, and a typical wind speed of 2.4 m/s is considered.

**Figure 37:**
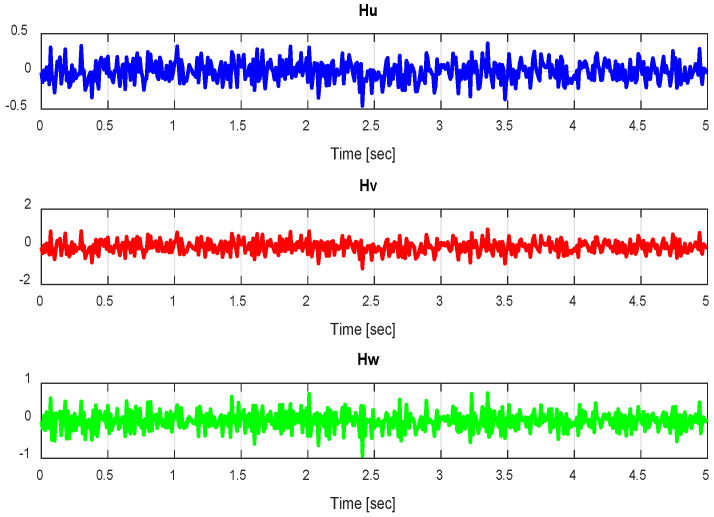
Wind velocity for scenario 2.

The figures (Figure 37) presented below relate to scenario 2.

**Figure 38:**
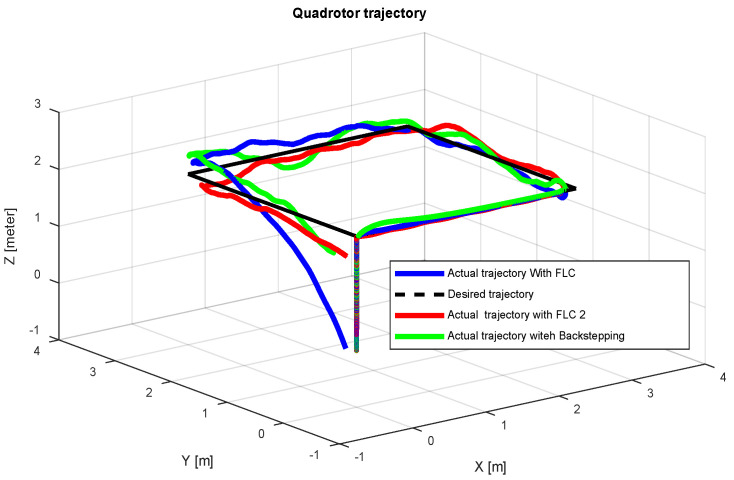
Quadrotor trajectory for scenario 2.

**Figure 39:**
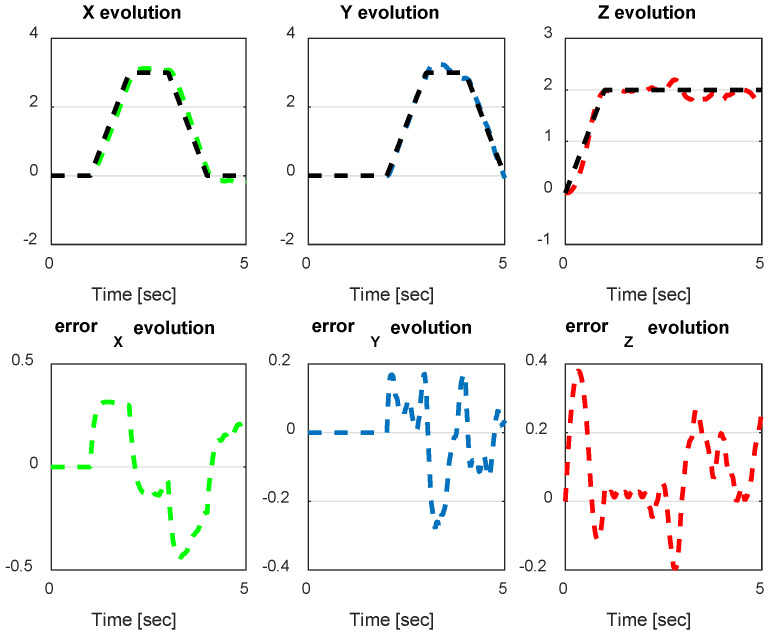
The errors of X, Y, and Z for PID-Type-2 FLC scenario 2.

**Figure 40:**
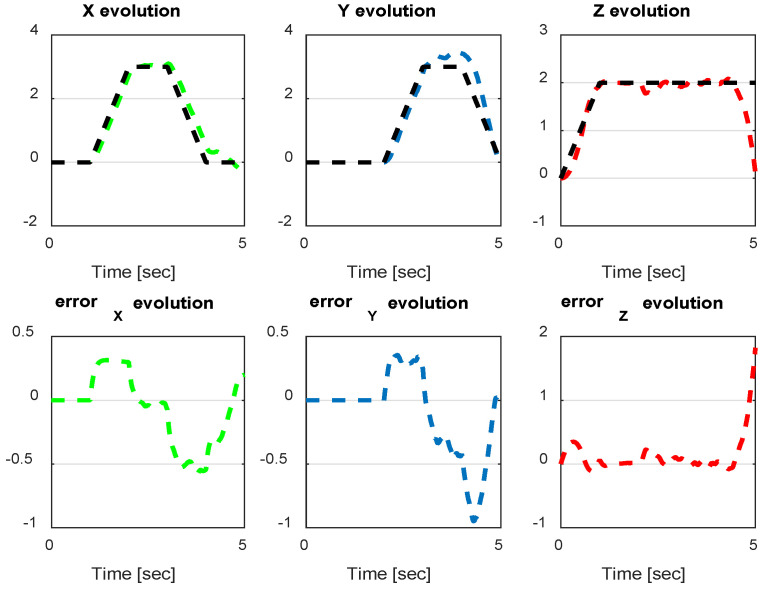
The errors of X, Y, and Z for PID FLC scenario 2.

**Figure 41:**
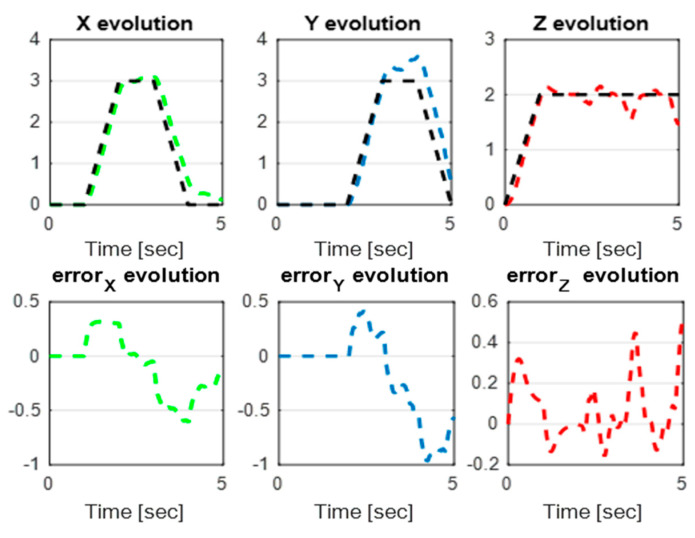
The errors of X, Y, and Z for Backstepping controller scenario 2.

The comparison between the three controllers, Backstepping, PID-Type-1 FLC and PID-Type-2 FLC, with and without GA tuning, is illustrated in Table 6. The Root Mean Square Errors (RMSEs) are compared for the same trajectory (Figure 27, Figure 36 and Figure 38) using several scenarios. As illustrated in Table 6, good precision is obtained by the optimal controller (PID-Type-2 FLC), even with the presence of significant wind disturbance. This result is justified by the robustness of the Type-2 FLC and optimality of the Genetic Algorithm. According to the results illustrated in Figure 38, the Type-2 FLC provides accurate tracking of the desired trajectory compared to the Backstepping controller and Type-1 FLC. This result is confirmed by Figure 39, Figure 40 and Figure 41, where the proposed approach provides the best accuracy.

## 6. Conclusions

The present research investigated the nonlinear system quadrotor aerial vehicle’s trajectory tracking issue. Three types of controllers were implemented, validated, and compared. First, a model-based approach using a Backstepping controller; second, a model-free control based on a PID FLC controller; and third, a PID-Type-2 FLC. The latter is validated, firstly, using heuristic PID parameters and, secondly, using optimal PID parameters based on GA. The proposed controllers PID FLC and GA-PID-Type-2 FLC are compared on the same trajectory. Good precision was obtained by the proposed PID-Type-2 FLC. Furthermore, the proposed controller showed more robustness in the face of wind disturbances and parameter uncertainties compared to the other approaches.

## Figures and Tables

**Table 1:** Quadrotor rule base.

		Height				
e		NB	N	Z	P	PB
de						
N		GUM	GD	GD	S	GU
Z		GDM	GD	S	GU	GUM
P		GD	S	GU	GUM	GUM

Where: N: Negative, Z: Zero, P: Positive, GUM: Go Up Much, GU: Go Up, S: Stand, GDM: Go Down Much, GD: Go Down, NB: Negative Big, PB: Positive Big.

**Table 2:** Parameters for output fuzzy controller.

**Variable**	GDM	GD	S	GU	GUM
**Output**	−10	−8	0	8	10

**Table 3:** Comparison of GA and other optimization approaches.

Criteria	Genetic Algorithm	Hybrid Spiral-Bacterial Foraging	Hybrid Grey Wolf-Whale Optimization
**Advantages**			
Global Search Capability	✓	✓	✓
Adaptability to Complex Systems	✓	✓	✓
Multi-Objective Optimization	✓	✓	✓
Empirical Optimization	✓	✓	✓
Offline Optimization	✓	✓	✓
Solution Diversity	✓	✓	✓
**Disadvantages**			
Computational Intensity	✓	✓ (Varies)	✓ (Varies)
Parameter Tuning	✓	✓ (Varies)	✓ (Varies)
No Guarantee for Global Optimality	✓	✓	✓
Real-Time Applicability for FLC	✓	✓	✓

Note that the checkmarks (✓) indicate whether the respective algorithm has an advantage or disadvantage for a specific criterion. The “Varies” notation indicates that the performance may depend on specific implementations or problem instances.

**Table 4:** Description of the quadrotor parameters [1].

Symbol	Description	Value
$I_{x}$	Moment of inertia X axis	0.007 kgm^2^
$I_{y}$	Moment of inertia Y axis	0.007 kgm^2^
$I_{z}$	Moment of inertia Z axis	0.012 kgm^2^
$J_{r}$	Rotor moment of inertia	6.5 × 10^−5^ kgm^2^
b	Thrust factor	4.13 × 10^−5^ Ns^2^
d	Drag factor	8.5 × 10^−7^ Nms^2^
l	Distance to the center of the quadrotor	0.17 m
m	Masse of quadrotor	0.68 kg
g	Gravitation constant	9.81 m/s^2^

**Table 5:** Parameters Results with GA optimization.

Controller	Parameters	PID-Type-1 FLC	PID-Type-2 FLC
Z	K1 K2 K4	18.20 11.98 0.47	13.8 3.98 0.6
φ	K5 K6	2 1.5	2.24 2.86
θ	K9 K10	2 1.4	0.19 66.42
ψ	K13 K14	2 15.1	−1.79 5.87

**Table 6:** Parameters for outputs of the proposed controllers (RMSE).

Controllers	Approaches	Z	Y
Backstepping	Without wind	2.17	4.29
	Scenario 1	2.32	5.84
	Scenario 2	3.69	9.50
PID-Type-1 FLC	Without wind	3.05	4.39
	GA Optimization	2.10	4.29
	Scenario 1	2.19	6.31
	Scenario 2	6.87	7.65
PID-Type-2 FLC	Without wind	3.00	4.29
	GA Optimization	2.30	3.90
	Scenario 1	2.43	3.90
	Scenario 2	3.25	4.16

## Data Availability

Data are contained within the article.
